# Bilateral associated game: Gain and loss in revaluation

**DOI:** 10.1371/journal.pone.0254218

**Published:** 2021-07-07

**Authors:** Wenna Wang

**Affiliations:** Department of Financial Mathematics, Xi’an University of Finance and Economics, Xi’an, Shaanxi, China; Utrecht University, NETHERLANDS

## Abstract

Hamiache introduces associated game to revalue each coalition’s worth, in which every coalition redefines his worth based on his own ability and the possible surpluses cooperating with other players. However, as every coin has two sides, revaluation may also bring some possible losses. In this paper, bilateral associated game will be presented by taking into account the possible surpluses and losses when revaluing the worth of a coalition. Based on different bilateral associated games, associated consistency is applied to characterize the equal allocation of non-separable costs value (EANS value) and the center-of-gravity of imputation-set value (CIS value). The Jordan normal form approach is the pivotal technique to accomplish the most important proof.

## Introduction

For any game, players may elaborate the game’s expectations and be willing to revalue their payments in accordance with these new expectations. Hamiache [1] initially introduces to us the idea of *associated game*, which is a modified game, for each coalition revaluing its worth in terms of a rule related to the original game. Hamiache assumes that every coalition considers itself at the center of a star-like graph and the players out of the coalition as isolated elements, and they may believe that the appropriation of at least a part of the possible surpluses, generated by its cooperation with each of the isolated players, is within reach.

A single-valued solution (i.e., value) is *associated consistent* if it gives the same payments to players in the original game as it does to players in the associated game. Hamiache [1] shows that the Shapley value is the unique solution that satisfies associated consistency, continuity and the inessential game property, which is called as Hamiache’s axiom system.

On the basis of Hamiache’s associated game, Hwang [2] modifies the definition of the possible surpluses to build an associated game for the EANS value. Although associated consistency and continuity are kept in these characterizations, they don’t stay within the axiom system of Hamiache. Xu et al. [3] considers the possible surpluses according to the sharing of the non-separable cost, and propose another type of associated game to axiomatize the EANS value within Hamiache’s axiom system. By the duality between the EANS value and the CIS value, the associated game is derived straightly for characterizing the CIS value. Later, Hwang [4] provides a new type of associated game generated from the idea of “union negotiations” for the axiomatization of the EANS value. Xu et al. [5] also constructs a new associated game by a pessimistic self-evaluation of worths of coalitions to characterize the CIS value.

Nevertheless, there does not exist any type of associated game that points out the negative effect, the risk that a coalition is kicked out of the grand coalition, which may be caused when the coalition overestimates its worth, and which might bring losses to the coalition. This motivates us to build an alternative type of associated game, named as bilateral associated game, by taking consideration of each coalition’s possible surpluses when being in the grand coalition and the possible losses when leaving the grand coalition. The corresponding associated consistency is applied to axiomatize the EANS value with continuity and the inessential game property. The similar characterization is also provided for the CIS value.

In Hamiache’s framework, he establishes the sequence of repeated associated games and proves that the sequence converges to an inessential game. The convergency is the key point for using associated consistency to axiomatize a value. Xu et al. [6] and Hamiache [7] put forward matrix analysis to show that the matrix expression of each associated linear transformation is diagonalizable. The matrix approach is also adopted to process this characterization by Hwang and Wang [8]. Béal et al. [9], afterwards, propose the Jordan normal form approach, which shortens the proofs of convergence as well as the axiomatic characterization by choosing a suitable basis for the space of TU cooperative games. This approach is put into use by Wang et al. [10] in the axiomatization of the egalitarian Shapley values. In the underlying article, the Jordan normal form is also employed as the pivotal technique to accomplish the proof.

The outline of the paper is as follows. Section 2 presents some preliminaries on cooperative game theory. In section 3, we introduce the definitions of bilateral associated games, and characterize two dual values by associated consistency with respect to different bilateral associated games. Section 4 shows the proof that the sequences of repeated bilateral associated games is convergent by the Jordan normal form approach.

## Preliminaries

Let *N* = {1, 2, ⋯, *n*} be a finite and fixed set. An element *i* ∈ *N* and a subset *S* ⊆ *N* are called a *player* and *coalition*, respectively. For each nonempty coalition *S* ⊆ *N*, its cardinality will be denoted as *s*. Denote by Ω_*N*_ the collection of all nonempty coalitions of *N*, i.e., Ω_*N*_ = {*S*|*S* ⊆ *N*, *S* ≠ ∅}.

A *cooperative game* with transferable utility (TU) is a pair 〈*N*, *v*〉, where $v:2^{N}\to R$ is the *characteristic function* assigning to each coalition *S* ∈ Ω_*N*_ the *worth* *v*(*S*), with the convention that *v*(∅) = 0. Denote by $G^{N}$ the game space consisting of all these TU games with player set *N*. For a given $\langle N,v\rangle \in G^{N}$, its *dual game* 〈*N*, *v*^*D*^〉 is defined by *v*^*D*^(*S*) = *v*(*N*) − *v*(*N*\*S*) for all *S* ⊆ *N*. A game $\langle N,v\rangle \in G^{N}$ is *almost inessential*, if for all *S* ⊊ *N*, *v*(*S*) = ∑_*i*∈*S*_ *v*({*i*}). A game $\langle N,v\rangle \in G^{N}$ is *inessential*, if for all *S* ∈ Ω_*N*_, it holds that *v*(*S*) = ∑_*i*∈*S*_ *v*({*i*}). Denote by $I^{N}$ the set of inessential games. A game $\langle N,v\rangle \in G^{N}$ is *constant*, if there is a a∈R such that, for each *S* ∈ Ω_*N*_, *v*(*S*) = *a*. Denote by $C^{N}$ the set of constant games.

The standard basis for $G^{N}$ is the ordered collection of games $S={\left(\langle N,\delta_{T}\rangle \right)}_{T\in \Omega_{N}}$, where the *standard game* $\langle N,\delta_{T}\rangle \in S$ is defined as: for each *S* ⊆ *N*, *δ*_*T*_(*S*) = 1 if *S* = *T*, and *δ*_*T*_(*S*) = 0 if *S* ≠ *T*. Another well-used basis for $G^{N}$ is the ordered collection of unanimity games $U={\left(\langle N,u_{T}\rangle \right)}_{T\in \Omega_{N}}$, where the *unanimity game* $\langle N,u_{T}\rangle \in U$ is defined as: for each *S* ⊆ *N*, *u*_*T*_(*S*) = 1 if *S* ⊇ *T*, and *u*_*T*_(*S*) = 0 if *S* ⊉ *T*. Notice that $I^{N}$ is a *n*-dimensional subspace of $G^{N}$, the ordered collection of singular unanimity games $I={\left(\langle N,u_{T}\rangle \right)}_{T\in \Omega_{N},t=1}$ forms a basis for $I^{N}$. $C^{N}$ is a one-dimensional subspace of $G^{N}$, and the game $\langle N,c\rangle \in C^{N}$ defined as for each *S* ∈ Ω_*N*_, *c*(*S*) = 1, constitutes a basis for $C^{N}$.

The solution part of cooperative game theory is to distribute the “fruits” of cooperation among the players. A single-valued solution, called *value* on $G^{N}$ is a function *ϕ* that assigns a single payoff vector $\phi \left(N,v\right)={\left(\phi_{i}\left(N,v\right)\right)}_{i\in N}\in R^{n}$ to every game $\langle N,v\rangle \in G^{N}$. The so-called payoff *ϕ*_*i*_(*N*, *v*) of player *i* in the game 〈*N*, *v*〉 represents an assessment by *i* of his gains for participating in the game.

For any game $\langle N,v\rangle \in G^{N}$, the well-known *Shapley value* is defined by Shapley [11] as

$$\begin{array}{c} Sh_{i}\left(N,v\right)=\sum_{S\subseteq N,S∍i}\frac{(n-s)!(s-1)!}{n!}\left[v(S)-v(S\backslash \{i\})\right],\;i\in N. \end{array}$$

The *EANS value*, introduced by Moulin [12], is given as

$$\begin{array}{c} EANS_{i}\left(N,v\right)=SC_{i}\left(N,v\right)+\frac{1}{n}[v\left(N\right)-\sum_{j\in N}SC_{j}\left(N,v\right)],\;i\in N, \end{array}$$

where *SC*_*j*_(*N*, *v*) = *v*(*N*) − *v*(*N* \ {*j*}) means the separable cost and the EANS value refers to all players should share the nonseparable cost *v*(*N*) − ∑_*j*∈*N*_ *SC*_*j*_(*N*, *v*) equally. Driessen and Funaki [13] define the *CIS value* as

$$\begin{array}{c} CIS_{i}\left(N,v\right)=v\left(\left\{i\right\}\right)+\frac{1}{n}[v\left(N\right)-\sum_{j\in N}v\left(\left\{j\right\}\right)],\;i\in N, \end{array}$$

which assigns to every player its individual worth, and distributes the remainder of the worth of the grand coalition *N* equally among all players.

Obviously, *EANS*(*N*, *v*) = *CIS*(*N*, *v*^*D*^) for all $\langle N,v\rangle \in G^{N}$ by the definition of dual game, *v*^*D*^({*j*}) = *SC*_*j*_(*N*, *v*) for all *j* ∈ *N*. So the EANS value and the CIS value are dual.

For any game $\langle N,v\rangle \in G^{N}$, two players *i*, *j* ∈ *N* are *symmetric* if, for every coalition *S* ⊆ *N* \ {*i*, *j*}, *v*(*S* ∪ {*i*}) = *v*(*S* ∪ {*j*}). Let $\phi :G^{N}\to R^{n}$ be a value, we consider the following properties.

- *Efficiency*: For any game $\langle N,v\rangle \in G^{N}$, ∑_*i*∈*N*_ *ϕ*_*i*_(*N*, *v*) = *v*(*N*).
- *Symmetry*: For any game $\langle N,v\rangle \in G^{N}$, if players *i*, *j* ∈ *N* are symmetric, then *ϕ*_*i*_(*N*, *v*) = *ϕ*_*j*_(*N*, *v*).
- *Translation covariance*: For any game $\langle N,v\rangle \in G^{N}$ and $x\in R^{n}$, *ϕ*(*N*, *v* + *x*) = *ϕ*(*N*, *v*) + *x*, where 〈*N*, *v* + *x*〉 is given by (*v* + *x*)(*S*) = *v*(*S*) + ∑_*i*∈*S*_ *x*_*i*_, for all *S* ⊆ *N*.
- *Inessential game property*: For any inessential game $\langle N,v\rangle \in I^{N}$, the value verifies *ϕ*_*i*_(*N*, *v*) = *v*({*i*})for all *i* ∈ *N*.
- *Continuity*: For every convergent sequence of games ${\left\{\langle N,v_{k}\rangle \right\}}_{k=0}^{\infty }$ and its limit game $\langle N,\tilde{v}\rangle$, we have $\lim_{k\to \infty }\phi \left(N,v_{k}\right)=\phi \left(N,\tilde{v}\right)$.

Except for these classical properties, associated consistency, originating from Hamiache, will be the main property applying in this article.

- *Associated consistency with respect to $\langle N,v_{\lambda }^{*}\rangle$*: For any game $\langle N,v\rangle \in G^{N}$ and its associated game $\langle N,v_{\lambda }^{*}\rangle$, the value satisfies $\phi \left(N,v\right)=\phi \left(N,v_{\lambda }^{*}\right)$.

Given any game $\langle N,v\rangle \in G^{N}$ and λ∈R, Hamiache [1] defines its *associated game* $\langle N,v_{\lambda }^{\text{Sh}}\rangle$ as follows: for all *S* ∈ Ω_*N*_,

$$\begin{array}{c} v_{\lambda }^{\text{Sh}}\left(S\right)≔v\left(S\right)+\lambda \sum_{j\in N\backslash S}[v(S\cup \{j\})-v(S)-v(\{j\})]. \end{array}$$ (1)

The associated game is interpreted by Hamiache as, any coalition *S* considers itself at the center of a star-like graph and the players in *N*\*S* as isolated elements. The coalition *S* believes that they may get λ percentage of the surpluses [*v*(*S* ∪ {*j*}) − *v*(*S*) − *v*({*j*})] generated by its cooperation with each of the isolated players.

Hamiache [1] shows that the Shapley value satisfies associated consistency with associated game $\langle N,v_{\lambda }^{\text{Sh}}\rangle$, and the sequence of repeated associated games converges to an inessential game. Therefore, he characterizes the Shapley value as the unique solution verifying associated consistency with respect to $\langle N,v_{\lambda }^{Sh}\rangle$, continuity and the inessential game property.

Hwang [2] modifies the definition of Hamiache’s associated game and defines $\langle N,v_{\lambda }^{\text{Hw}}\rangle$, for all *S* ∈ Ω_*N*_,

$$\begin{array}{c} v_{\lambda }^{\text{Hw}}\left(S\right)=v\left(S\right)+\lambda \sum_{j\in N\backslash S}[v\left(S\cup \left\{j\right\}\right)-v\left(S\right)-SC_{j}\left(N,v\right)]. \end{array}$$ (2)

Hwang explains that the coalition *S* expects to get λ(λ∈R) percentage of the surpluses [*v*(*S* ∪ {*j*}) − *v*(*S*) − *SC*_*j*_(*N*, *v*)] generated by its cooperation with each of the isolated players *j* ∈ *N*\*S*. Since the corresponding sequence of repeated associated games doesn’t converge to an inessential game, but to the sum of an inessential game and a constant game, Hwang replaces the inessential game property be efficiency, symmetry and translation covariance.

Xu et al. [5] introduce the almost inessential game (property) to the solution part of cooperative game theory.

- *Almost inessential game property*: For any almost inessential game 〈*N*, *v*〉, the value satisfies *ϕ*_*i*_(*N*, *v*) = *v*({*i*}) + *α*[*v*(*N*) − ∑_*j*∈*N*_ *v*(*j*)] for all *i* ∈ *N*, where *α* ∈ [0, 1].

With the observation of almost inessential game, each (*n* − 1)-person game (with players in *N*) is an inessential game, so each player deserves his individual value. When we consider the *n*-person game by dividing *v*(*N*), all players should have equal rights on what is a surplus or a deficit induced by cooperation.

Xu et al. [5] define an *associated game* $\langle N,v_{\lambda }^{\text{Xu}}\rangle$ as

$$\begin{array}{c} v_{\lambda }^{\text{Xu}}\left(S\right)=\{\begin{array}{cc} v\left(S\right)+\lambda \sum_{j\in S}[v(S)-v(S\backslash \{j\})-v(\{j\})], & S⊊N;\\ v(N), & S=N. \end{array} \end{array}$$ (3)

Different from Hamiache [1] and Hwang [2], Xu et al. interpret that the associated game is considered as an adaptation of a given game such that it reflects a pessimistic self-evaluation of worths of coalitions. Although the CIS value satisfies associated consistency with respect to $\langle N,v_{\lambda }^{\text{Xu}}\rangle$, the corresponding sequence of repeated associated games converges to an almost inessential game. Hence, Xu et al. replaces Hamiache’s axiom system with associated consistency, continuity, the almost inessential game property and efficiency.

In favor of continuing Hamiache’s axiom system, Xu et al. [3] define the associated game $\langle N,v_{\lambda }^{E}\rangle$ as, for all *S* ∈ Ω_*N*_,

$$\begin{array}{c} v_{\lambda }^{E}\left(S\right)=v\left(S\right)+\lambda \{\frac{s}{n}[v\left(N\right)-\sum_{j\in N}SC_{j}\left(N,v\right)]-[v\left(S\right)-\sum_{j\in S}SC_{j}\left(N,v\right)]\}. \end{array}$$ (4)

In this associated game, the coalition *S* takes account of the fact that, besides its worth *v*(*S*), it will get a share in the nonseparable costs. With respect to $\langle N,v_{\lambda }^{E}\rangle$, the EANS value is characterized by associated consistency, continuity and the inessential game property.

Xu et al. [3], replacing in $\langle N,v_{\lambda }^{E}\rangle$ the term *SC*_*j*_(*N*, *v*) by its dual worth *v*({*j*}), also define the associated game $\langle N,v_{\lambda }^{C}\rangle$, for all *S* ∈ Ω_*N*_, as

$$\begin{array}{c} v_{\lambda }^{C}\left(S\right)=v\left(S\right)+\lambda \{\frac{s}{n}[v\left(N\right)-\sum_{j\in N}v\left(\left\{j\right\}\right)]-[v\left(S\right)-\sum_{j\in S}v\left(\left\{j\right\}\right)]\} \end{array}$$ (5)

to characterize the CIS value.

As described above, no matter Hamiache and Hwang consider a coalition as the center of the cooperation, or Xu et al. give some new self-evaluations, in all of these associated games, every coalition wants to achieve more gain. Whereas, as the proverb goes: grasp all, lose all. Therefore, we make our efforts to pursue a more appropriate way to revalue coalition’s worth in the next section.

## Bilateral associated games

When a coalition overvalues its worth, it must take the risk of being removed from the grand coalition, which was ignored in these previous associated games. Underneath, we introduce a new type of associated game, named as bilateral associated game, to revalue the worths of coalitions. This bilateral associated game includes, in a coalition’s worth, the possible surpluses after forming the grand coalition and the possible losses after leaving the grand coalition, when they estimate their own ability.

**Definition 1**. *Given any game* $\langle N,v\rangle \in G^{N}$ *and* λ∈R, *define its* E-*bilateral associated game* $\langle N,v_{\lambda }^{E}\rangle$ *as*, *for all S* ∈ Ω_*N*_,

$$\begin{array}{cccc} v_{\lambda }^{E}\left(S\right) & = & v(S) & \text{(6a)}\\ & + & \lambda \sum_{j\in N\backslash S}[v(S\cup \{j\})-v(S)-v(\{j\})] & \text{(6b)}\\ & + & \lambda \{[v\left(S\right)-\sum_{j\in S}v\left(\left\{j\right\}\right)]-\frac{s}{n}[v\left(N\right)-\sum_{j\in N}v\left(\left\{j\right\}\right)]\}. & \text{(6c)} \end{array}$$

The worth of coalition *S* in this bilateral associated game has the following elements:

(6a) *v*(*S*) is the elementary payoff that the coalition *S* can get explicitly on its own.
(6b) When the grand coalition *N* is formed, the coalition *S* regards itself as the center and believes that it can get part of the possible surpluses [*v*(*S* ∪ {*j*}) − *v*(*S*) − *v*({*j*})] shown in (6b), which is generated by the cooperation between the coalition *S* and the players out of *S* individually.
(6c) The coalition *S* regards the possible losses as the difference between the surplus [*v*(*S*) − ∑_*j*∈*S*_ *v*({*j*})] by supposing that they leave the grand coalition and the loss by distributing the surplus [*v*(*N*) − ∑_*j*∈*N*_ *v*({*j*})] generated by cooperation equally among themselves when they are in the grand coalition, listed as (6c). The coalition *S* must also undertake the partial losses.

**Remark 1**. Notice that, in the E-bilateral associated game, the method of revaluing coalition’s worth is the combination of Hamiache’s associated game (see Eq (1)) for the Shapley value and Xu’s second associated game (see Eq (5)) for the CIS value.

**Lemma 1**. *The EANS value satisfies associated consistency with respect to the* E-*bilateral associated game* $\langle N,v_{\lambda }^{E}\rangle$.

**Proof**. Following the definition of the associated game $\langle N,v_{\lambda }^{E}\rangle$, it is easy to get that $v_{\lambda }^{E}\left(N\right)=v\left(N\right)$ and for any *i* ∈ *N*,

$$\begin{array}{cc} v_{\lambda }^{E}\left(N\backslash \left\{i\right\}\right) & =v\left(N\backslash \left\{i\right\}\right)+\lambda \{[v\left(N\backslash \left\{i\right\}\right)-\sum_{j\in N\backslash \{i\}}v\left(\left\{j\right\}\right)]\\ & \;\;+[v(N)-v(N\backslash \{i\})-v(\{i\})]-\frac{n-1}{n}[v\left(N\right)-\sum_{j\in N}v\left(\left\{j\right\}\right)]\}\\ & =v\left(N\backslash \left\{i\right\}\right)+\frac{\lambda }{n}[v\left(N\right)-\sum_{j\in N}v\left(\left\{j\right\}\right)], \end{array}$$

then

$$\begin{array}{ccc} SC_{i}\left(N,v_{\lambda }^{E}\right) & = & v_{\lambda }^{E}\left(N\right)-v_{\lambda }^{E}\left(N\backslash \left\{i\right\}\right)\\ & = & SC_{i}\left(N,v\right)-\frac{\lambda }{n}[v\left(N\right)-\sum_{j\in N}v\left(\left\{j\right\}\right)], \end{array}$$

where *SC*_*i*_(*N*, *v*) = *v*(*N*) − *v*(*N* \ {*i*}), so we have

$$\begin{array}{ccc} EANS_{i}\left(N,v_{\lambda }^{E}\right) & = & SC_{i}\left(N,v_{\lambda }^{E}\right)+\frac{1}{n}[v_{\lambda }^{E}\left(N\right)-\sum_{j\in N}SC_{j}\left(N,v_{\lambda }^{E}\right)]\\ & = & SC_{i}\left(N,v\right)-\frac{\lambda }{n}[v\left(N\right)-\sum_{j\in N}v\left(\left\{j\right\}\right)]\\ & & +\frac{1}{n}\{v\left(N\right)-\sum_{j\in N}[SC_{j}\left(N,v\right)-\frac{\lambda }{n}\left[v\left(N\right)-\sum_{k\in N}v\left(\left\{k\right\}\right)\right]]\}\\ & = & SC_{i}\left(N,v\right)+\frac{1}{n}[v\left(N\right)-\sum_{j\in N}SC_{j}\left(N,v\right)]\\ & = & EANS_{i}\left(N,v\right). \end{array}$$

This completes the proof.

In light of the dual relationship between the CIS value and the EANS value, we also construct C-bilateral associated game for the CIS value as follows.

**Definition 2**. *Given any game* $\langle N,v\rangle \in G^{N}$ *and* λ∈R, *define its* C-*bilateral associated game* $\langle N,v_{\lambda }^{C}\rangle$ *as*, *for all S* ∈ Ω_*N*_,

$$\begin{array}{cccc} v_{\lambda }^{C}\left(S\right) & = & v(S) & \text{(7a)}\\ & + & \lambda \sum_{j\in S}\{[v(N)-v(N\backslash \{j\})]-[v(S)-v(S\backslash \{j\})]\} & \text{(7b)}\\ & + & \lambda \{[v\left(S\right)-\sum_{j\in S}SC_{j}\left(N,v\right)]-\frac{s}{n}[v\left(N\right)-\sum_{j\in N}SC_{j}\left(N,v\right)]\}. & \text{(7c)} \end{array}$$

The three parts respectively are: the elementary payoff of coalition *S*, the possible surpluses and the possible losses. Particularly, (7b) supposes that, for a coalition *S*, the surplus per member is measured by the loss of the overall marginal contribution [*v*(*N*) − *v*(*N*\{*j*})] over the coalitional marginal contribution [*v*(*S*) − *v*(*S*\{*j*})] of the number *j* ∈ *S*, when they are the participants of the grand coalition *N*.

According to the definitions of the E-bilateral associated game and the C-bilateral associated game, they have the following relationship.

**Proposition 1**. *For any game* $\langle N,v\rangle \in G^{N}$, $\langle N,{\left(v_{\lambda }^{E}\right)}^{D}\rangle =\langle N,{\left(v^{D}\right)}_{\lambda }^{C}\rangle$.

**Proof**. For any game $\langle N,v\rangle \in G^{N}$, it is obvious that $v_{\lambda }^{E}\left(N\right)=v\left(N\right)$ and $v_{\lambda }^{C}\left(N\right)=v\left(N\right)$ by (6) and (7). Further, for all *S* ⊆ *N*, we have

$$\begin{array}{ccc} & & {\left(v_{\lambda }^{E}\right)}^{D}\left(S\right)\\ & = & v_{\lambda }^{E}\left(N\right)-v_{\lambda }^{E}\left(N\backslash S\right)\\ & = & v\left(N\right)-v\left(N\backslash S\right)-\lambda \sum_{j\in S}[v(N\backslash S\cup \{j\})-v(N\backslash S)-v(\{j\})]\\ & & -\lambda ([v\left(N\backslash S\right)-\sum_{j\in N\backslash S}v\left(\left\{j\right\}\right)]-\frac{n-s}{n}[v\left(N\right)-\sum_{j\in N}v\left(\left\{j\right\}\right)])\\ & = & v(N)-v(N\backslash S)\\ & & -\lambda \sum_{j\in S}([v(N)-v(N\backslash S)]-[v(N)-v(N\backslash S\cup \{j\})]-v\left(\left\{j\right\}\right))\\ & & -\lambda ([v\left(N\backslash S\right)-\sum_{j\in N\backslash S}v\left(\left\{j\right\}\right)]-[v\left(N\right)-\sum_{j\in N}v\left(\left\{j\right\}\right)]+\frac{s}{n}[v\left(N\right)-\sum_{j\in N}v\left(\left\{j\right\}\right)])\\ & = & v^{D}\left(S\right)+\lambda \sum_{j\in S}([v^{D}\left(N\right)-v^{D}\left(N\backslash \left\{j\right\}\right)]-[v^{D}\left(S\right)-v^{D}\left(S\backslash \left\{j\right\}\right)])\\ & & +\lambda ([v^{D}\left(S\right)-\sum_{j\in S}SC_{j}\left(N,v^{D}\right)]-\frac{s}{n}[v^{D}\left(N\right)-\sum_{j\in N}SC_{j}\left(N,v^{D}\right)])\\ & = & {\left(v^{D}\right)}_{\lambda }^{C}\left(S\right). \end{array}$$

This shows that $\langle N,{\left(v_{\lambda }^{E}\right)}^{D}\rangle =\langle N,{\left(v^{D}\right)}_{\lambda }^{C}\rangle$.

According to Lemma 1, Proposition 1, and the fact that the CIS value and the EANS value are each other’s dual, it follows that the CIS value satisfies associated consistency with the C-bilateral associated game.

**Lemma 2**. *The CIS value satisfies associated consistency with respect to the* C-*bilateral associated game* $\langle N,v_{\lambda }^{C}\rangle$.

For any game $\langle N,v\rangle \in G^{N}$ and its associated game $\langle N,v_{\lambda }^{Sh}\rangle$, in the framework of Hamiache’s characterization process, the sequence of repeated associated games ${\left\{\langle N,v_{\lambda }^{m*Sh}\rangle \right\}}_{m=0}^{\infty }$ plays a pivotal role. The term of order *m* in the sequence is the associated game of the term of order (*m* − 1) and the term of order 0 is the original game 〈*N*, *v*〉. Next, we present the result of convergence of the two sequences of repeated bilateral associated games in Definition 1 and Definition 2, which will be proved in the next section by the Jordan normal form approach.

**Lemma 3**. *For any game* $\langle N,v\rangle \in G^{N}$ *and* $0<\lambda <\frac{2}{n-1}$, *the sequences of repeated bilateral associated games* ${\left\{\langle N,v_{\lambda }^{m*E}\rangle \right\}}_{m=0}^{\infty }$ *and* ${\left\{\langle N,v_{\lambda }^{m*C}\rangle \right\}}_{m=0}^{\infty }$ *converge, and the two limit games are both inessential*.

For the convergent sequence of repeated bilateral associated games, according to associated consistency and continuity, the value *ϕ*(*N*, *v*) is equal to the value $\phi (N,\tilde{v})$ of the limit game $\langle N,\tilde{v}\rangle$. As the limit games of two sequences of repeated bilateral associated games are both inessential, by the inessential game property, $\phi \left(N,v\right)=\phi \left(N,\tilde{v}\right)$ is identified with the worths of all singleton coalitions.

**Theorem 1**. *For* $0<\lambda <\frac{2}{n-1}$, *the EANS value is the unique value satisfying the inessential game property, continuity, and associated consistency with respect to* E-*bilateral associated game* $\langle N,v_{\lambda }^{E}\rangle$.

**Theorem 2**. *For* $0<\lambda <\frac{2}{n-1}$, *the CIS value is the unique value satisfying the inessential game property, continuity, and associated consistency with respect to* C-*bilateral associated game* $\langle N,v_{\lambda }^{C}\rangle$.

Hwang [2] characterizes the EANS value as the unique value satisfying associated consistency, continuity, efficiency, symmetry and translation covariance. Using the bilateral associated game in Definition 1 (resp. Definition 2), we will weaken Hwang’s axioms to characterize the EANS value (resp. the CIS value) as replacing efficiency and symmetry by the zero game property. A game 〈*N*, *v*〉 is called *zero game*, if *v*(*S*) = 0 for all *S* ⊆ *N*.

- *Zero game property*: For any zero game 〈*N*, *v*〉, the solution verifies *ϕ*_*i*_(*N*, *v*) = 0 for all *i* ∈ *N*.

Apparently, efficiency together with symmetry implies the zero game property, but not vice versa.

**Theorem 3**. *For* $0<\lambda <\frac{2}{n-1}$, *the EANS value (resp. the CIS value) is the unique value satisfying the zero game property, translation covariance, continuity, and associated consistency with respect to* E-*bilateral associated game* $\langle N,v_{\lambda }^{E}\rangle$ *(resp*. C-*bilateral associated game* $\langle N,v_{\lambda }^{C}\rangle$).

**Proof**. Compared with the axiom systems in Theorem 1 and Theorem 2, the inessential game property is replaced by the zero game property and translation covariance. Lemma 3 says that the limit games of the sequences of repeat bilateral associated games ${\left\{\langle N,v_{\lambda }^{m*E}\rangle \right\}}_{m=0}^{\infty }$ and ${\left\{\langle N,v_{\lambda }^{m*C}\rangle \right\}}_{m=0}^{\infty }$ are inessential. Therefore, it is sufficient to check that on the class of inessential games, a value satisfying the inessential game property is equivalent to satisfying the zero game property and translation covariance.

Let *ϕ* be a value on $G^{N}$ satisfying the inessential game property. For any inessential game $\langle N,v\rangle \in I^{N}$, it can be written as, for all *S* ⊆ *N*,

$$\begin{array}{c} v\left(S\right)=w\left(S\right)+\sum_{i\in S}x_{i}, \end{array}$$

where *w*(*S*) = 0 for all *S* ⊆ *N* and *x*_*i*_ = *v*({*i*}) for all *i* ∈ *N*.

Since the zero game 〈*N*, *w*〉 is also inessential, by the inessential game property of *ϕ*, we have *ϕ*_*i*_(*N*, *w*) = 0 for all *i* ∈ *N*. This implies the zero game property of *ϕ*. Considering the inessential game property of *ϕ* on 〈*N*, *v*〉, for *i* ∈ *N*,

$$\begin{array}{c} \phi_{i}\left(N,v\right)=v\left(\left\{i\right\}\right)=\phi_{i}\left(N,w\right)+x_{i}. \end{array}$$

This implies that the value *ϕ* satisfies translation covariance. And the equivalence on the contrary is trivial.

## The proof of Lemma 3 by the Jordan normal form approach

Drawing on Hamiache’s viewpoint, the sequence of the repeated associated games being convergent for sufficiently small λ, plays a main role in the proof of characterizations with associated consistency. Béal et al. [9] propose the Jordan normal form approach, and point that the Jordan normal form of the associated transformation matrix can be quickly computed, which is effective to shorten the proof of convergence as well as the axiomatic characterization by choosing a suitable basis for the vector space of TU games. Deriving from the Jordan normal form of a linear transformation, Béal et al. put forward the following corollary.

**Corollary 1** (Béal et al. [9]). *Let V be a finite dimensional real vector space of dimension n and let* Ψ *be a linear transformation* Ψ: *V* → *V*. *Assume that the following conditions hold*.

1. *1 is an eigenvalue of* Ψ *and the multiplicity of 1 is equal to the dimension of its eigenspace*.
2. *Each other eigenvalue ε of* Ψ *is such that* |*ε*| < 1.

*Then, for any* $\langle N,v\rangle \in G^{N}$ , *we have* ${\left(\Psi^{m}\left(v\right)\right)}_{m\in N}\overset{m\to +\infty }{\to }v^{*}$, where v* *belongs to the eigenspace associated with the eigenvalue 1*.

Béal et al. [9] introduce that the *Jordan normal form approach* can be exposed as follows:

1. Choose a basis $B=(b_{1},\cdots ,b_{2^{n}-1})$ for $G^{N}$.
2. For each linear transformation Ψ and for each *l* ∈ {1, ⋯, 2^*n*^ − 1}, compute the image Ψ(*b*_*l*_).
3. From step 2, it can be easy to express the matrix of Ψ with respect to B and conclude that the matrix is upper triangular. So the entries on the main diagonal are the eigenvalues of Ψ.
4. It needs to verify that the conditions 1 and 2 in the Corollary 1 are satisfied, which ensures the convergence result of the linear transformation.

According to these steps, it is necessary to choose a suitable basis for TU games. In the proof of the sequence of repeated E-bilateral associated games, we choose the ordered collection $B^{1}=\left(I,c,S_{n-2}\right)$ constitutes a basis (the proof is similar to the proof of Proposition 3 in [9]) for $G^{N}$, where $I={\left(\langle N,u_{T}\rangle \right)}_{T\in \Omega_{N},t=1}$, $S_{n-2}={\left(\langle N,\delta_{T}\rangle \right)}_{T\in \Omega_{N},t\leq n-2}$ and 〈*N*, *c*〉 is a basis for the set of all constant games.

For convenience, it’s necessary to define a linear transformation ${ϒ}_{\lambda }^{E}:G^{N}\to G^{N}$ as follows: for any *S* ∈ Ω_*N*_ and λ∈R,

$$\begin{array}{c} {ϒ}_{\lambda }^{E}\left(v\right)\left(S\right)=v_{\lambda }^{E}\left(S\right). \end{array}$$

**Proposition 2**. *Consider the basis* $B^{1}$ *for* $G^{N}$. *The following facts hold*.

1. *For each i* ∈ *N*, ${ϒ}_{\lambda }^{E}\left(u_{\left\{i\right\}}\right)=u_{\left\{i\right\}}$.
2.
${ϒ}_{\lambda }^{E}\left(c\right)=\left[1-\left(n-1\right)\lambda \right]c+\frac{(n-1)\lambda }{n}\sum_{i\in N}u_{\left\{i\right\}}$ .
3. *For each k* ∈ *N*,
  $$\begin{array}{c} {ϒ}_{\lambda }^{E}\left(\delta_{\left\{k\right\}}\right)=\left[1-\left(n-2\right)\lambda \right]\delta_{\left\{k\right\}}-\lambda c+\frac{\lambda }{n}\sum_{i\in N}u_{\left\{i\right\}}. \end{array}$$
4. *For T* ∈ Ω_*N*_, 2 ≤ *t* ≤ *n* − 2,
  $$\begin{array}{c} {ϒ}_{\lambda }^{E}\left(\delta_{T}\right)=\left[1-\left(n-t-1\right)\lambda \right]\delta_{T}+\lambda \sum_{j\in T}\delta_{T\backslash \{j\}}. \end{array}$$

**Proof**. **1**. According to the expression (6) of E-bilateral associated game, it is obvious that for each *i* ∈ *N*, ${ϒ}_{\lambda }^{E}\left(u_{\left\{i\right\}}\right)=u_{\left\{i\right\}}$.

**2**. For any *S* ∈ Ω_*N*_,

$$\begin{array}{ccc} {ϒ}_{\lambda }^{E}\left(c\right)\left(S\right) & = & c\left(S\right)+\lambda \sum_{j\in N\backslash S}[c(S\cup \{j\})-c(S)-c(\{j\})]\\ & & +\lambda \{[c\left(S\right)-\sum_{j\in S}c\left(\left\{j\right\}\right)]-\frac{s}{n}[c\left(N\right)-\sum_{j\in N}c\left(\left\{j\right\}\right)]\}\\ & = & \left[1-\left(n-s-1\right)\lambda \right]c\left(S\right)+\lambda \sum_{j\in N\backslash S}c\left(S\cup \left\{j\right\}\right)\\ & & -\frac{s\lambda }{n}c\left(N\right)-\frac{(n-s)\lambda }{n}\sum_{j\in N}c\left(\left\{j\right\}\right)\\ & = & \left[1-\left(n-1\right)\lambda \right]+\frac{(n-1)\lambda }{n}s. \end{array}$$

Therefore,

$$\begin{array}{c} {ϒ}_{\lambda }^{E}\left(c\right)=\left[1-\left(n-1\right)\lambda \right]c+\frac{(n-1)\lambda }{n}\sum_{i\in N}u_{\left\{i\right\}}. \end{array}$$

**3**. For each *k* ∈ *N* and any *S* ∈ Ω_*N*_,

$$\begin{array}{ccc} & & {ϒ}_{\lambda }^{E}\left(\delta_{\left\{k\right\}}\right)\left(S\right)\\ & = & \delta_{\left\{k\right\}}\left(S\right)+\lambda \sum_{j\in N\backslash S}[\delta_{\left\{k\right\}}\left(S\cup \left\{j\right\}\right)-\delta_{\left\{k\right\}}\left(S\right)-\delta_{\left\{k\right\}}\left(\left\{j\right\}\right)]\\ & & +\lambda \{[\delta_{\left\{k\right\}}\left(S\right)-\sum_{j\in S}\delta_{\left\{k\right\}}\left(\left\{j\right\}\right)]-\frac{s}{n}[\delta_{\left\{k\right\}}\left(N\right)-\sum_{j\in N}\delta_{\left\{k\right\}}\left(\left\{j\right\}\right)]\}\\ & = & \left[1-\left(n-s-1\right)\lambda \right]\delta_{\left\{k\right\}}\left(S\right)-\lambda +\frac{s\lambda }{n}. \end{array}$$

Therefore,

$$\begin{array}{c} {ϒ}_{\lambda }^{E}\left(\delta_{\left\{k\right\}}\right)=\left[1-\left(n-2\right)\lambda \right]\delta_{\left\{k\right\}}-\lambda c+\frac{\lambda }{n}\sum_{i\in N}u_{\left\{i\right\}}. \end{array}$$

**4**. For *T* ∈ Ω_*N*_, 2 ≤ *t* ≤ *n* − 2, and any *S* ∈ Ω_*N*_,

$$\begin{array}{ccc} & & {ϒ}_{\lambda }^{E}\left(\delta_{T}\right)\left(S\right)\\ & = & \delta_{T}\left(S\right)+\lambda \sum_{j\in N\backslash S}[\delta_{T}\left(S\cup \left\{j\right\}\right)-\delta_{T}\left(S\right)-\delta_{T}\left(\left\{j\right\}\right)]\\ & & +\lambda \{[\delta_{T}\left(S\right)-\sum_{j\in S}\delta_{T}\left(\left\{j\right\}\right)]-\frac{s}{n}[\delta_{T}\left(N\right)-\sum_{j\in N}\delta_{T}\left(\left\{j\right\}\right)]\}\\ & = & \left[1-\left(n-s-1\right)\lambda \right]\delta_{T}\left(S\right)+\lambda \sum_{j\in N\backslash S}\delta_{T}\left(S\cup \left\{j\right\}\right).\\ & = & \{\begin{array}{ll} 1-(n-t-1)\lambda , & \text{if}\;S=T;\\ \lambda , & \text{if}\;S=T\backslash \{j\},j\in T;\\ 0, & \text{otherwise}. \end{array} \end{array}$$

Therefore,

$$\begin{array}{c} {ϒ}_{\lambda }^{E}\left(\delta_{T}\right)=\left[1-\left(n-t-1\right)\lambda \right]\delta_{T}+\lambda \sum_{j\in T}\delta_{T\backslash \{j\}}. \end{array}$$

This completes the proof.

In the proof of the sequence of repeated C-bilateral associated games, we choose the ordered collection $B^{2}=\left(I,S_{2}^{\prime }\right)$ constitutes a basis (the proof is similar to the proof of Proposition 3 in [9]) for $G^{N}$, where $S_{2}^{\prime }$ is the inverse order collection of $S_{2}={\left(\langle N,\delta_{T}\rangle \right)}_{T\in \Omega_{N},t\geq 2}$.

The linear transformations ${ϒ}_{\lambda }^{C}:G^{N}\to G^{N}$ is defined as follows: for any *S* ∈ Ω_*N*_ and λ∈R,

$$\begin{array}{c} {ϒ}_{\lambda }^{C}\left(v\right)\left(S\right)=v_{\lambda }^{C}\left(S\right). \end{array}$$

**Proposition 3**. *Consider the basis* $B^{2}$ *for* $G^{N}$. *The following facts hold*.

1. *For each i* ∈ *N*, ${ϒ}_{\lambda }^{C}\left(u_{\left\{i\right\}}\right)=u_{\left\{i\right\}}$.
2.
${ϒ}_{\lambda }^{C}\left(\delta_{N}\right)=\left[1-\left(n-1\right)\lambda \right]\delta_{N}+\frac{(n-1)\lambda }{n}\sum_{i\in N}u_{\left\{i\right\}}$ .
3. *For T* ∈ Ω_*N*_, *t* = *n* − 1,
  $$\begin{array}{c} {ϒ}_{\lambda }^{C}\left(\delta_{T}\right)=\left[1-\left(t-1\right)\lambda \right]\delta_{T}+\lambda \delta_{N}-\frac{\lambda }{n}\sum_{i\in N}u_{\left\{i\right\}}. \end{array}$$
4. *For T* ∈ Ω_*N*_, 2 ≤ *t* ≤ *n* − 2,
  $$\begin{array}{c} {ϒ}_{\lambda }^{C}\left(\delta_{T}\right)=\left[1-\left(t-1\right)\lambda \right]\delta_{T}+\lambda \sum_{j\in N\backslash T}\delta_{T\cup \{j\}}. \end{array}$$

The proof of Proposition 3 is similar to the proof of Proposition 2 and so it is omitted.

**Proof of Lemma 3**.

From Proposition 2, the matrix of ${ϒ}_{\lambda }^{E}$ with respect to the basis $B^{1}$ is lower triangular. And the matrix of ${ϒ}_{\lambda }^{C}$ is also lower triangular, which is based on the basis $B^{2}$ and Proposition 3. These also make it easy to know that their eigenvalues are both 1 with multiplicity *n*, and 1 − (*t* − 1)λ, (*t* = 2, 3, ⋯, *n*) with multiplicity ${(}_{t}^{n})$. Now, we show that conditions 1 and 2 in Corollary 1 are satisfied. Denote by ${ϒ}_{\lambda }≔\left\{{ϒ}_{\lambda }^{E},{ϒ}_{\lambda }^{C}\right\}$,

1.
$I={\left(\langle N,u_{T}\rangle \right)}_{T\in \Omega_{N},t=1}$ is a basis for $I^{N}$. By Proposition 2 (1) and Proposition 3 (1), it can be directly deduced that 1 is an eigenvalue of ϒ_λ_ and the multiplicity of 1 is *n* equaling to the dimension of its eigenspace $I^{N}$.
2. For any *t* ∈ {2, 3, ⋯, *n*}, when $0<\lambda <\frac{2}{n-1}$, it holds that |1 − (*t* − 1)λ| < 1.

According to Corollary 1, we know that for any $\langle N,v\rangle \in G^{N}$,

$$\begin{array}{c} {\left({ϒ}_{\lambda }^{m}\left(v\right)\right)}_{m\in N}\overset{m\to +\infty }{\to }\tilde{v}, \end{array}$$

where $\langle N,\tilde{v}\rangle$ belongs to $I^{N}$ and indeed is an inessential game. This completes the proof of Lemma 3.

## Conclusion

When Hamiache introduced the associated game as a way to revalue coalition’s worth, he considered that every coalition revalues its worth based on its own ability and the possible surpluses cooperating with other players. However, every coin has two sides. Revaluation can bring not only some possible extra gain, but also the risk that coalition may be kicked off the grand coalition, regarded as the possible losses. The bilateral associated game took into account the two sides, including the possible surpluses and the possible losses in the process of revaluation. Different methods that describe the possible surpluses and losses can define different bilateral associated games for different values.

We built bilateral associated games respectively for the EANS value and the CIS value. The corresponding sequences of repeated bilateral associated games were proved to be converging to inessential game by the Jordan normal form approach. According to the two bilateral associated games, associated consistency is taken to characterize the EANS value and the CIS value with continuity and the inessential game property. In addition, the zero game property and translation covariance, instead of the inessential game property, are also used to characterize the EANS value and the CIS value.

## References

[1] Hamiache G. Associated consistency and Shapley value. International Journal of Game Theory 2001 Sept; 30: 279–289. doi: 10.1007/s001820100080

[2] Hwang Y. Associated consistency and equal allocation of nonseparable costs. Economic Theory 2006 Aug; 28: 709–719. doi: 10.1007/s00199-005-0640-7

[3] Xu G, van den Brink R, van der Laan G, Sun H. Associated consistency characterization of two linear values for TU games by matrix approach. Linear Algebra and its Applications 2015 Apr; 471: 224–240. doi: 10.1016/j.laa.2014.12.036

[4] Hwang Y, Julia R, Ismail R. Union negotiations: Complement-associated games. Operations Research Letters 2017 Jan; 45: 126–132. doi: 10.1016/j.orl.2017.01.003

[5] Xu G, Wang W, Dong H. Axiomatization for the center-of-gravity of imputation set value. Linear Algebra and its Applications 2013 Oct; 439(8): 2205–2215. doi: 10.1016/j.laa.2013.06.026

[6] Xu G, Driessen TSH, Sun H. Matrix analysis for associated consistency in cooperative game theory. Linear Algebra and its Applications 2008 Apr; 428(7): 1571–1586. doi: 10.1016/j.laa.2007.10.002

[7] Hamiache G. A matrix approach to the associated consistency with an application to the Shapley value. International Game Theory Review 2010; 12(2): 175–187. doi: 10.1142/S0219198910002581

[8] Hwang Y, Wang B. A matrix approach to the associated consistency with respect to the equal allocation of non-separable costs. Operations Research Letters 2016 Oct; 44: 826–830. doi: 10.1016/j.orl.2016.10.008

[9] Béal S, Rémila E, Solal P. Characterizations of three linear values for TU games by associated consistency: simple proofs using the Jordan normal form. International Game Theory Review 2016 Mar; 18(1): 1–21.

[10] Wang W, Sun H, Xu G, Hou D. Procedural interpretation and associated consistency for the egalitarian Shapley values. Operations Research Letters 2017 Feb; 45: 164–169. doi: 10.1016/j.orl.2017.01.012

[11] Shapley LS. A value for N-person games. in: Kuhn H.W., Tucker A.W. (Eds.), Contributions to the theory of games II, Princeton University Press, Princeton 1953; 307–317.

[12] Moulin H. The separability axiom and equal sharing method. Journal of Economic Theory 1985; 36: 120–148. doi: 10.1016/0022-0531(85)90082-1

[13] Driessen TSH, Funaki F. Coincidence of and collinearity between game theoretic solutions. OR Spektrum 1991 Mar; 13: 15–30. doi: 10.1007/BF01719767

